# Impact of Integrated Amrita Meditation Technique on Adrenaline and Cortisol Levels in Healthy Volunteers

**DOI:** 10.1155/2011/379645

**Published:** 2011-01-20

**Authors:** Balakrishnan Vandana, Kannan Vaidyanathan, Lakshmiy Ammal Saraswathy, Karimassery Ramaiyer Sundaram, Harish Kumar

**Affiliations:** ^1^Department of Physiology, Amrita Institute of Medical Sciences, Amrita Lane, Ponekkara P.O., Cochin 682 041, Kerala, India; ^2^Department of Biochemistry, Amrita Institute of Medical Sciences, Amrita Lane, Ponekkara P.O., Cochin 682 041, Kerala, India; ^3^Department of Biostatistics, Amrita Institute of Medical Sciences, Amrita Lane, Ponekkara P.O., Cochin 682 041, Kerala, India; ^4^Department of Endocrinology, Amrita Institute of Medical Sciences, Amrita Lane, Ponekkara P.O., Cochin 682 041, Kerala, India

## Abstract

The objective was to find out the effect of Integrated Amrita Meditation Technique (IAM) on the stress hormones: adrenaline and cortisol. One hundred and fifty healthy subjects were randomized into three groups. Blood was collected at 0 hour, 48 hours, 2 months, and 8 months after the first visit. Adrenaline was analyzed by ELISA and cortisol by Chemiluminescent method. In the IAM, PMR and control groups 44, 44, and 36 came, respectively, for the baseline visit. Within group, cortisol and adrenaline levels reduced in the IAM 48 hours onwards and the fall sustained until 8 months (*P* < .05). ANCOVA (Repeated measures) on adrenaline taking the four levels of observation showed a highly significant (*P* = .001) drop in the IAM group. The mean cortisol values between groups were not statistically significant (*P* = .138). IAM Technique was effective in reducing adrenaline and cortisol levels within group comparisons.

## 1. Introduction

Selye was one of the first to study and attempt to understand the impact of stress on the body [1]. He defined “stress” as the nonspecific response of the body to any demand. Stress occurs when expectations do not match the situations in life. Selye considered the Hypothalamo Pituitary Adrenal (HPA) axis and genetic factors as the effectors of stress response [2]. An immediate response of body to stress occurs through the sympathetic Adreno-Medullary pathway (Active Coping), and the long-term response to stress occurs through the Adreno-Cortical pathway (Passive Coping) [3]. 

Irrespective of the mechanism involved in the response to stress, a healthy diet, regular exercise, reduction of negative thoughts through open discussions, and relaxation techniques are some of the methods by which stress can be controlled. Among the widely used relaxation techniques, yoga and meditation are now gaining popularity in the West as well as the East as effective tools to develop a more positive attitude and thus keep stress in check. By simple definition, meditation is a complex process involving changes in cognition, sensory perception, release of hormones, and autonomic activity [4]. 

The first written description of meditation comes from The Mahabharata, a sixth-century BC epic written by Vyasa, which contains the Bhagavad-Gita in which the “Dhyana Yoga” [5], a form of meditation, is recommended as a technique to sooth the mind. After that, Patanjali produced the classical yoga text—The Yoga Sutras [6]. The address of Swami Vivekanada in 1893 at the Parliament of Religions in Chicago and “The Autobiography of a Yogi” by Yogananda [7] introduced the concepts of yoga and meditation to the West. The Transcendental Meditation Technique initiated by Maharishi Mahesh Yogi gained popularity in the West in 1960s and the first papers of meditation research on the physiological benefits of meditation were published in early 1970s [8]. The practice of meditation is expected to improve both physical and mental health and also contribute to psychological well-being. There are a large number of scientific papers on TM which have looked at the physiological effects and also catecholamine in relation to meditation [9]. 

Stress questionnaires, electromyography, autonomic Nervous System tests, and hormonal analyses are the frequently used research techniques to assess stress. The autonomic stability was compared between meditators and nonmeditators in 1973 through simple galvanic skin response and meditators were found to have a better response [10]. The value of Alternative Medicine (meditation, meditative prayer, yoga and relaxation response) in reducing stress-related symptoms has been reported to be effective and the authors had recommended meditation over prescription of drugs as the preferred treatment for mild hypertension in 1984 [11]. The practice of Transcendental Meditation was found to reduce blood pressure in patients with Hypertension [12, 13] and also improve other risk factors like dyslipidemia and Insulin Resistance, thus having an overall impact of reducing cardiovascular risk in patients with Metabolic Syndrome who are at high risk for developing coronary artery disease [14, 15]. Both exercise and meditation can improve psychological health, but when these modalities were compared, meditation was found to be superior not only in improving psychological health but also reducing endocrine stress markers [16]. 

It is recognized that many different meditation techniques have widely differing methodologies [17]. A meta-analysis of over 600 studies indicates that not all techniques have the same effects and in most of the studies, small sample size, suboptimal control groups, lack of long-term followup, and problems of adherence among participants were the factors criticized [18–20]. The aim of our study was to analyze the effect of Integrated Amrita Meditation Technique on the stress hormones in healthy subjects, with a view to understand its potential benefit on both the Active and Passive coping pathways.

IAM is a new meditation technique. The hypothesis of our study is that IAM lowers stress hormones. So the objective of the current study is to find out whether this new technique is effective in reducing adrenaline and cortisol levels in healthy volunteers.

## 2. Materials and Methods

### 2.1. Subjects

 One hundred and fifty college students from two different colleges (age 18–21 years) were recruited for the study. Any student who had not undergone any specialized relaxation training and had volunteered to participate was included. Chronic smokers, alcoholics, and psychiatric patients were excluded through a screening questionnaire.

 The subjects were randomly assigned three groups by the lottery method. They were numbered and tokens were prepared with these numbers. From this the tokens were drawn out and the subjects were assigned to three different groups. The first group was trained to practice a meditation technique called Integrated Amrita Meditation (IAM) Technique. The subjects randomized to the second group were given training on Progressive Muscle Relaxation (PMR) technique, the third group served as controls and did not practice any meditation technique or do any relaxation exercises.

### 2.2. Integrated Amrita Meditation Technique

This is a simple combination of yoga, pranayama, and meditation. The technique was designed and presented to the world by Her Holiness Mata Amritanandamayi Devi. It is a simple meditation technique suited for an ordinary urban person. It consists of energizing exercises (yogic postures) for up to 8 minutes, a brief period of relaxation for 2 minutes, and 13 minutes of meditation. At the end of the technique, the subjects are asked to remain in silence for 5 minutes.

 The components of IAM Technique are as follows.

Relaxation exercises/yogic postures: which progressively relax the muscles and joints and so the mind too. It also has an energizing, holistic effect. Breathing exercises (focused breathing): which draw attention to the way one breathes and prompt a more complete breathing. Awareness: throughout the process awareness is the main component. One is encouraged to be aware of all the subtleties of each of the steps. One part in particular focuses on the flow of breath. Visualization: focusing the mind on an internal point, rather than on a physical object outside. Criteria for successful practice: belief in a spiritual master and chanting the mantra given by the master is recommended. Only the first class is guided. The later practice is without the help of any external means. 

There were periodic refresher courses. IAM Technique was taught by teachers who were well versed with the technique and approved as teachers by the Mata Amritanandamayi Math. The relaxation of the mind by this technique is expected to remove stress and expand thinking in general, making the subject more creative. Subjects in this group practiced this technique once daily and compliance was assessed by self-maintained diary.

### 2.3. Progressive Muscle Relaxation Technique

It is a technique of stress management developed by American physician Edmund Jacobson in the early 1920s. Progressive muscle relaxation is based upon the practice of tensing or tightening one muscle group at a time followed by a relaxation phase with release of the tension. PMR involves a physical and mental component. With the eyes closed and in a sequential pattern, a tension is given to a muscle group purposefully for approximately 10 seconds and then released for 20 seconds before continuing with the next muscle group. The whole PMR session takes approximately 30 minutes [21]. A trained physiotherapist taught the PMR Technique.

All subjects were followed up for a total duration of eight months. A self-maintained diary assessed compliance in both the groups. All the subjects in the IAM and PMR groups continued regular practice of these techniques throughout the eight-month period. Practicing the technique minimum four times a week was taken as the standard of compliance. 

Institutional Ethics Committee clearance was obtained and subjects signed informed consent forms prior to participation in the study. Blood samples were collected at baseline (before any intervention), and again 48 hours later. Subsequently blood samples were collected 2 months and eight months after randomization. All samples were collected at 8 AM.

### 2.4. Adrenaline Analysis

Plasma adrenaline analysis was done by ELISA (Adrenaline EIA). Adrenaline was extracted using a cis-diol-specific affinity gel. It was then acylated to N-acyladrenaline and converted enzymatically to N-acylmetanephrine, acylated adrenaline from the sample and solid phase of the micro titer plate compete for antiserum binding sites. The antibody bound to solid phase was detected by an anti rabbit IgG peroxidase conjugate using a suitable substrate. The reaction was monitored at 450 nm.

### 2.5. Cortisol Assay

Cortisol was estimated by Chemiluminiscent micro particle immunoassay method. The sample and anti cortisol coated paramagnetic micro particles are combined to create a reaction mixture. Cortisol present in the sample binds to the anti cortisol coated micro particles. After incubation cortisol acridinum labeled conjugate is added to the reaction mixture and trigger solutions are added to the reaction mixture. The resulting chemiluminescent reaction is measured as Relative Light Units (RLU).

### 2.6. Statistical Analysis

The sample size was calculated from the available information in published papers on meditation [22]. Minimum sample size was estimated as 25 in each of the three groups (95% confidence and 80% power). Since we anticipated a 50% drop out rate on the basis of previous studies, we decided to recruit 150 subjects. The data was analyzed using SPSS-Version 11 (SPSS Inc, Rostock, IL) statistical package. Since both adrenaline and cortisol did not follow normal distribution and were heterogeneous between the groups and also because of the smaller sample size, all the analyses were done on the logarithm of the values of the variables. The within group comparison was done by paired *t*-test. 

When the within-group analysis is done with all the time points together, only the minimum number of cases observed at a particular point will be considered at all points, reducing the sample size at each point to very small. Also, replacement of the missing values by imputation method, especially when there are many missing values may not be very valid, as imputation method itself has a lot of criticism. Hence the within-group comparison has been done for the groups separately for time points 1 and 2, 1 and 3, 1 and 4 so that the maximum number of cases available can be considered for the analysis. The difference in the mean values of adrenaline and cortisol between groups was done by applying ANCOVA (repeated measures) taking four levels of observation (visits 1, 2, 3, and 4).

## 3. Results

The one hundred and fifty volunteers were randomized into the three groups, namely, IAM, PMR, and control. However, some of the subjects dropped out of the study after signing the Informed Consent Document, and forty-four subjects each attended the initial training at baseline visit in the IAM and PMR group and 36 subjects in the control group came for the baseline visit (visit 1). The compliance was found to be 85.7% in IAM and 84.4% in PMR group. The groups were found to be comparable on basis of age, sex, and education (Table 1).

In the IAM group, analysis of the mean adrenaline values within the same group compared to visit 1 by paired *t*-test showed significant drop in the second visit, and the decrease was sustained in all the subsequent visits. In the PMR group, there was a significant drop at the 48-hour visit, but at the two months' and eight months' visit, there was no difference from the baseline level. The control group did not show any significant difference in any of the visits (Table 2). 

When the difference in the mean value adrenaline between the three groups taking the four levels of observation was analyzed by the ANCOVA (Repeated measures), it was found to be highly significant (*P* = .001). On doing the above analysis on pairs of groups, the difference in the mean value of IAM was found to be significantly lower than PMR and control groups (IAM- PMR- *P* = .001, IAM-control- *P* = .001). The mean adrenaline values on applying ANCOVA (Repeated measures) taking four levels of observation (visits 1, 2, 3, 4) did not show a significant linear trend (*P* = .473) but the quadratic trend was close to significance (*P* = .066). 

Analysis of the mean cortisol values within the IAM group by paired *t*-test showed a statistically significant decrease in the cortisol levels from visit 1 to visit 2 (48 hours). The cortisol levels at visit 3 (two months) and visit 4 (8 months) also showed a statistically significant decrease compared to the baseline visit (Table 3). In the PMR group, there was no difference in cortisol levels at visit 2 when compared to the baseline; however, visit 3 and visit 4 did show a statistically significant drop compared to visit 1. The control group did not show any significant reduction in the cortisol levels until visit 4 (Table 3).

On applying ANCOVA (Repeated measures) taking four levels of observation (visits 1, 2, 3, and 4), it was seen that the difference in the mean values of cortisol among the groups was not significant (*P* = .506) but it showed a significant linear trend (*P* = < .001).

## 4. Discussion

The Integrated Amrita Meditation Technique is a combination of simple breathing and muscle relaxation exercises with deep meditation. In addition to a control group who had no intervention, we included a group trained to practice a simple muscle relaxation technique in order to compare the impact of IAM with the control group but also with the PMR group, to see whether the benefits if any, were due to simple exercises or whether the meditation along with the exercises provided additional benefit. We had planned the follow-up visits at 48 hours, 2 months, and 8 months with a view to assessing the immediate impact, and also the intermediate and long-term sustained effects of meditation. One of the limitations of the current study is that we had selected students of a particular age group who may not be representing the whole population. But this was done keeping in mind that a long-term followup was planned for the study and also the strict assessment of compliance was feasible only with a group of student volunteers who could be contacted over a regular period of time. Still we found that the drop-out rate was high because many of the students were busy with their academic activities and missed some visits. Drop out of subjects was there in all the three groups. 

In our study, we found that the mean adrenaline levels declined in the IAM group immediately after IAM (48 hours) and this was sustained until 8 months. Adrenaline levels were significantly lower than control and PMR groups immediately after meditation (48 hours) and also at 2- and 8-month followup visits, suggesting that the drop in adrenaline levels following meditation was immediate and sustained up to 8 months.

Initial studies on Transcendental meditation had looked at urinary catecholamines and urinary VMA measurements [23]. The disadvantage of measuring urinary catecholamine levels is that kidney disorders and metabolic defects can affect these measurements and they may be also influenced by diet and other factors like smoking which had not been considered in the study. Later the plasma adrenaline levels of people regularly doing TM were compared to the plasma adrenaline of those not doing TM, and a significant decrease was seen in the TM practitioners [24]. But these studies were just a comparison of adrenaline levels done randomly without any long-term followup. 

Exposure to stress causes an immediate increase in adrenaline through the Hypothalamo Pituitary Adrenal (HPA) pathway [25, 26]. Relaxation techniques like meditation are thought to reduce stress and thus lower adrenaline levels through the active stress coping pathway. The subjects who practiced IAM probably had decrease in stress response which was reflected in the lower values of adrenaline compared to controls. We had measured adrenaline levels at 48 hours after the onset of meditation to study the immediate impact of IAM on the stress hormone levels and our results indicate the same, as adrenaline levels dropped within 48 hours of onset of meditation.

Our results show that cortisol levels in the IAM group declined significantly from 48 hours itself and remained at significantly lower levels than the baseline visit, after two months and eight months of practice of IAM. In fact, there was a continuous decrease in the cortisol levels over time (Table 3) showing clearly that IAM had an immediate impact and lowered cortisol values by 48 hours after initiating meditation practice, and this benefit was sustained up to 8 months with steadily declining levels. The PMR group did not show any change at 48 hours, however cortisol levels did decline at 2 months and 8 months compared to baseline, suggesting that sustained practice of muscle relaxation exercises did have an effect of reducing cortisol levels over time. 

When the cortisol levels were analyzed in a study on Transcendental Meditation, the cortisol levels in 8 meditators and nonmeditators were compared before, during, and after a meditation session and there was a significant drop in the meditation group [27]. Walton et al. [28] and Mc Lean et al. [29, 30] have done several studies on the effect of TM on cortisol levels, and they found that the technique lowered cortisol in normal subjects and also postmenopausal women exposed to laboratory stressors. Serum cortisol levels were found to be significantly reduced in 52 males after the practice of Buddhist Dhammakaya Meditation in comparison with 30 nonmeditators [22]. The practice of Kinesthetic Meditation by young adults for a period of five weeks reported a significant lowering of salivary cortisol levels in comparison with the control group [31]. Salivary cortisol levels were found to be decreased in long-term practitioners of TAI Chi when compared to beginners [32]. 

Long-term exposure to stress increases cortisol levels and relaxation techniques are found to reduce cortisol through a corticotrophin releasing hormone modulated pathway. In our study, a trend towards a decrease in cortisol levels was seen only towards the eight months' visit when compared to PMR and control groups. In fact, our results suggest that the beneficial effects of cortisol reduction may be slow but lasting and although it did not reach statistical significance it showed a downward trend. A larger period of followup or larger sample size may be needed to conclusively show a statistically significant drop in cortisol levels after IAM practice.

## 5. Conclusion

 The significant fall in adrenaline levels and the downward trend seen in cortisol show the immediate and long-term efficacy of the IAM Technique in reducing the stress hormones.

## Figures and Tables

**Table 1:** Comparison of sociodemographic variables of the three groups.

Variable		IAM	PMR	Control	*P* value
Sex*	Male	18.2 (*n* = 8)	20.5 (*n* = 9)	16.7 (*n* = 6)	.908
	Female	81.8 (*n* = 36)	79.5 (*n* = 35)	83.3 (*n* = 30)	

Age* (years)	18-19	77.3 (*n* = 34)	77.3 (*n* = 34)	72.2 (*n* = 26)	.837
	20 and above	22.7 (*n* = 10)	22.7 (*n* = 10)	27.8 (*n* = 10)	

Education*	Completed school	13.6 (*n* = 6)	15.9 (*n* = 7)	22.2 (*n* = 8)	.823
	Degree	65.9 (*n* = 29)	63.6 (*n* = 28)	63.9 (*n* = 23)	
	Postgraduate	20.5 (*n* = 9)	20.5 (*n* = 9)	13.9 (*n* = 5)	

*Figures are percentages.

**Table 2:** Comparison of adrenaline levels from visit 1 to visit 4 within each group.

	Sample (*n*)	Mean ± SD (in picograms/dL)	*P* value in comparison with visit 1
		IAM	

Visit 1	36	132.47 ± 22.11	Change: 17.36
Visit 2	36	115.11 ± 19.55	.001*
Visit 1	30	130.50 ± 10.31	Change: 14.4
Visit 3	30	116.10 ± 22.61	.001*
Visit 1	18	127.22 ± 10.61	Change: 9.82
Visit 4	18	117.40 ± 11.61	.007*

PMR			

Visit 1	32	144.88 ± 23.86	Change: 14.41
Visit 2	32	130.47 ± 22.05	.025*
Visit 1	29	145.21 ± 23.48	Change: 12.45
Visit 3	29	132.76 ± 26.31	.084
Visit 1	21	143.38 ± 26.75	Change: .63
Visit 4	21	142.75 ± 12.71	.774

Control			

Visit 1	33	137.73 ± 18.12	Change: −1.85
Visit 2	33	139.58 ± 30.64	.997
Visit 1	28	138.01 ± 19.50	Change: −5.13
Visit 3	28	143.14 ± 20.34	.331
Visit 1	21	139.76 ± 20.08	Change: .37
Visit 4	21	139.39 ± 22.24	.906

*P value less than .05. Change = mean decrease.

**Table 3:** Comparison of cortisol levels from visit 1 to visit 4 within each group.

	Sample (*n*)	Mean ± SD (in micrograms/dL)	*P* value in comparison with visit 1
		IAM	

Visit 1	35	14.20 ± 4.45	Change: 2.39
Visit 2	35	11.81 ± 3.55	.002*
Visit 1	30	14.28 ± 4.5	Change: 3.45
Visit 3	30	10.83 ± 2.77	.001*
Visit 1	29	13.80 ± 4.62	Change: 5.3
Visit 4	29	8.5 ± 2.77	.001*

PMR			

Visit 1	32	13.71 ± 4.14	Change: 1.39
Visit 2	32	12.32 ± 4.75	.088
Visit 1	31	13.32 ± 4.21	Change: 2.33
Visit 3	31	10.99 ± 4.33	.014*
Visit 1	23	14.19 ± 4.33	Change: 3.55
Visit 4	23	10.64 ± 3.56	.007*

Control			

Visit 1	32	13.87 ± 5.2	Change: 1.2
Visit 2	32	12.67 ± 5.20	.081
Visit 1	28	13.84 ± 5.4	Change: 0.81
Visit 3	28	13.03 ± 5.41	.333
Visit 1	23	13.49 ± 5.6	Change: 2.25
Visit 4	23	11.24 ± 3.9	.004*

*P value less than .05. Change = mean decrease.
